# Photodynamic Inactivation of Bacteria in Boar Semen with Blue LED Light

**DOI:** 10.3390/microorganisms13030643

**Published:** 2025-03-12

**Authors:** Isabel Katharina Maaßen, Anne-Marie Luther, Mohammad Varzandeh, Steffen Hackbarth, Dagmar Waberski

**Affiliations:** 1 Unit for Reproductive Medicine/Clinic for Swine and Small Ruminants, University of Veterinary Medicine Hannover, 30559 Hannover, Germany; 2 Photobiophysics, Institute of Physics, Humboldt University of Berlin, 10117 Berlin, Germany

**Keywords:** photodynamic inactivation, boar semen, bacteria, antimicrobial resistance, semen preservation

## Abstract

The photodynamic inactivation (PDI) of bacteria is a promising alternative to antibiotics in boar semen extenders. It was recently established using the illumination of semen samples containing 2 µM of the photosensitizer 5,10,15,20-tetrakis(N-methyl-4-pyridyl)-21H,23H-porphine (TMPyP) with white LED light. High concentrations of TMPyP require strict sample handling in the dark to avoid uncontrolled photodynamic effects caused by ambient light. This study was designed to examine whether lower concentrations of PS could be utilized along with a narrow band blue LED light source, which aligns with TMPyP’s Soret band, thereby minimizing light-induced disruption. A dose-response study with blue LED light exposure of sperm revealed no light toxicity. Importantly, substituting the established white light PDI with blue light illumination and 0.5 µM TMPyP resulted in robust antimicrobial efficiency and sperm compatibility in long-term stored semen samples. This modification led to the confirmation of the hypothesis that a diminished TMPyP concentration in concert with blue LED light facilitates semen handling in normal laboratory light while avoiding unintended light effects. In conclusion, this study plays a pivotal role in augmenting the practicality of the innovative PDI technology by establishing a method that is less susceptible to unanticipated effects of ambient light during sample management.

## 1. Introduction

Artificial insemination (AI) is a widely used reproductive technology in domestic animal breeding. The preservation of semen for use in AI necessitates efficient antimicrobial control to hinder bacterial growth, thereby avoiding potential damage to sperm and preventing infections in the female reproductive system. However, the regular use of antibiotics in semen extenders has promoted multi-drug resistances, notably in porcine species [1], where the standard practice of semen storage at 17 °C readily facilitates bacterial growth [2].

**Figure 1:**
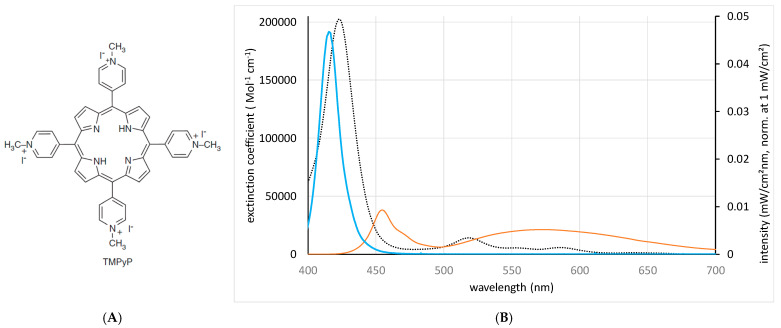
Structural formula of the photosensitizer TMPyP (**A**). Extinction spectra as determined for the photosensitizer TMPyP (dotted line) and emission spectra of the blue (blue line) and the white (red line) LED light source (**B**): the blue LED light overlaps with the Soret band of the TMPyP, while the broadband white light has limited coincidence with the photosensitizer’s absorption spectrum. The LED spectra are normalized for an overall intensity of 1 mW/cm² each. The extinction coefficients are in good agreement with those reported by [18].

Recently, photodynamic inactivation (PDI) technology emerged as an innovative alternative to eliminating bacteria in extended boar semen [3]. In essence, the PDI principle relies on a photosensitizer (PS) molecule that, when exposed to light, absorbs photons. This propels the PS into an excited triplet state [4]. Thereafter, the PS interacts with ambient ground state oxygen to produce singlet oxygen and other reactive oxygen species (ROS), thereby damaging biomolecules in microorganisms. The challenge with PDI application in semen is to inactivate bacteria while preserving sperm viability. In vitro findings, corroborated by in vivo fertility, demonstrated the successful use of the cationic PS 5,10,15,20-tetrakis(1-methylpyridinium-4-yl) porphyrin (TMPyP; Figure 1A), when used with white light LED illumination for 90 s [3]. The TMPyP was chosen as a PS based on its beneficial properties, including thermal and photochemical stability, environmentally friendly attributes [5], water solubility, cost-efficiency, and a high quantum yield of 0.77 ± 0.04 for singlet oxygen generation in water [6], thus offering a high bacterial killing efficacy [7]. Furthermore, TMPyP has demonstrated non-toxicity to boar spermatozoa [3] and efficacy against various bacteria [8]. Importantly, its extracellular activity precludes the emergence of antimicrobial resistance [3,9]. The highly positively charged molecule has an affinity to many, mostly negatively charged, microorganisms and is effective against a broad array of pathogens, including viruses, fungi, and protozoa [4,5].

Albeit promising, the application of PDI in AI necessitates further optimization. One issue with the currently established PDI white light method is the relatively high TMPyP concentration (2 µM), which can generate undesired PDI effects in ambient light, necessitating sample management in darkness. However, semen processing in AI labs invariably exposes the samples to ambient light, potentially risking sperm damage due to continuous, uncontrolled photodynamic reactions.

To circumvent this, we propose a novel approach that uses a lower TMPyP concentration. Taking into account that the electronic absorption spectra of the porphyrin TMPyP have an intensive Soret band of around 420 nm [10], we hypothesize that more illumination with LED light in this range would allow a reduction in the TMPyP concentration, without compromising the antimicrobial effectiveness. For this reason, we propose to replace the white LED light source (400–750 nm) with a narrow emission blue LED light of around 415 nm. This approach needs to consider that spermatozoa may react to blue light exposition in the absence of any PS. The biochemical response to light, or ‘Photobiomodulation’, is described in various cells, including spermatozoa [11], which possess endogenous PS in the mitochondria and plasma membrane [12,13]. While the exact mechanism is yet to be elucidated, the blue light-induced photoreaction in spermatozoa implicates several endogenous PS, such as cytochromes, heme molecules, flavins, porphyrins, pyridine cofactors, nicotinamide, adenine dinucleotides, and Fe–S clusters [11,14,15]. Blue light could exert both negative and positive impacts on spermatozoa depending on the wavelength (nm), power density (mW/cm^2^), exposition time (s, min), and pattern (illumination cycles with dark phases) [11,14,16,17]. The objective of this study was to explore whether substituting white LED light with blue LED light could enable a reduction in TMPyP concentration while maintaining antimicrobial effectiveness and sperm quality. Applying a reduced PS concentration could potentially permit the use of PDI methodology in semen laboratories under standard lighting conditions, thereby avoiding uncontrolled photoreactions after illumination.

## 2. Materials and Methods

### 2.1. Photosensitizer and Illumination Setup

The photosensitizer TMPyP was added to an 8 mL sample of extended semen, which was placed in transparent plastic bags (Quick Tip narrow type, Minitüb GmbH, Tiefenbach, Germany) measuring 5.2 × 7 cm, as has been utilized in previous studies [3]. The thickness of the sample in the central region of the bag was 5 mm. The PDI method was applied, using white LED light with a broad spectral emission (400–750 nm, PDI_white_; [3]), comparing it to a narrow emission blue LED light, characterized by an FWHM of 16 nm and centralized around 415 nm (PDI_blue_). The spectra of both the white and blue light illumination setups, along with the extinction spectrum of TMPyP are illustrated in Figure 1B. The intrinsic advantage of using blue light for excitation lies in its specific overlap with the Soret band of TMPyP. Assuming that the sample bag has a thickness of 5 mm, coupled with a TMPyP concentration of 0.5 µM, as was the case in this study, we can compare the number of absorbed photons, as outlined in Equation (1) [3], under both white and blue illumination at identical illumination intensities. The efficiency of photosensitization improved by a factor of 14 with blue excitation compared to white excitation, using identical overall intensity.

(1) $$n_{abs}=\frac{1}{hc}\int_{\lambda_{2}}^{\lambda_{1}}I(\lambda )∙(1-10^{-A\left(\lambda \right)})∙\lambda d\lambda$$

*I*(*λ*) is the intensity per cm^2^ and *A*(*λ*) denotes the absorbance of the TMPyP [3].

Comparable photosensitization is obtained when a sample containing 2 µM TMPyP is illuminated using 4.5 mW/cm² white LED light (400–750 nm) when compared to a sample containing 0.5 µM TMPyP and illuminated with 2.5 mW/cm² blue LED light (415 nm).

Experiments were carried out under red darkroom light (10 W COB LED with a maximum emission at 705 nm and a long pass filter RG695), in order to avoid the effects of background light [3].

### 2.2. Experimental Design

**Figure 2:**
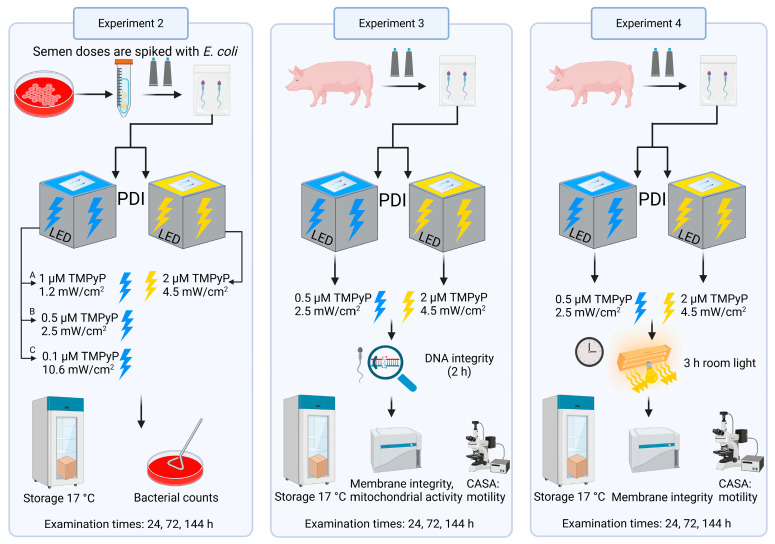
Overview of the designs of photodynamic inactivation (PDI) experiments (created with BioRender.com).

An overview of the experimental design is presented in Figure 2. Using extended boar semen, experiments were performed to assess the effect of blue LED light on sperm motility (Experiment 1), and to compare PDI_blue_ with PDI_white_ for their antimicrobial efficiency (Experiment 2) as well as sperm quality (Experiment 3). The novel PDI_blue_ method was then compared with control PDI_white_ [3] for their susceptibility to post-illumination light effect on preserved spermatozoa (Experiment 4).

#### 2.2.1. Experiment 1: Effect of Blue LED Light on Sperm Kinematics

The aim was to examine the effect of different doses of blue light on sperm kinematics during long-term semen storage. Subsamples of extended semen (n = 9) from three different boars were exposed to different intensities of blue LED light in the absence of TMPyP: 2.9 mW/cm^2^ for 60 s, 5.8 mW/cm^2^ for 90 s, 11.2 mW/cm^2^ for 90 s, 11.2 mW/cm^2^ for 3 × 180 s with 6 min dark intervals, 17.8 mW/cm^2^ for 60 s, 25.6 mW/cm^2^ for 60 s, and 32.7 mW/cm^2^ for 60 s. A further sample served as a dark control. Sperm motility was assessed at three time points during the subsequent semen storage: 2, 72, and 144 h (Section 2.6.1).

#### 2.2.2. Experiment 2: Antimicrobial Efficiency of PDI_blue_ Compared to PDI_white_

The aim was to examine the effect of different TMPyP concentrations in combination with different blue light intensities on antimicrobial efficiency. Semen from eight different boars was collected, and the bacterial species and counts were determined after culture in sheep blood agar (Section 2.5). Semen was then extended in the antibiotic-free long-term extender Androstar Premium (Minitüb GmbH, Tiefenbach, Germany) and spiked with *Escherichia coli* to 10^3^–10^4^ CFU/mL to enhance the bacterial load. Subsamples were subjected to PDI_blue_ with three different combinations of TMPyP concentrations and light intensities: 0.1 µM, 10.6 mW/cm^2^; 0.5 µM, 2.5 mW/cm^2^; and 1 µM, 1.2 mW/cm^2^. A further subsample was subjected to the previously established PDI_white_ protocol with 2 µM TMPyP and 4.5 mW/cm^2^ [3]. All PDI samples were illuminated for 90 s. Viability of bacteria was monitored in a control semen sample in the antibiotic-free BTS medium consisting of 205 mM glucose, 20.4 mM Na_3_C_6_H_5_O_7_, 10.0 mM KCl, 15 mM NaHCO_3_, and 3.36 mM EDTA [19]. At 24, 72, and 144 h semen storage, bacterial counts were determined (Section 2.5).

#### 2.2.3. Experiment 3: Sperm Quality After PDI_blue_ Compared to PDI_white_

The aim was to determine the influence of the effective antimicrobial PDI_blue_, as identified in Experiment 2, on sperm quality in comparison to the established PDI_white_ [3]. Semen from eight boars, extended in Androstar Premium, was subjected to PDI_blue_ with 0.5 µM TMPyP and 2.5 mW/cm^2^, and to PDI_white_ with 2 µM TMPyP and 4.5 mW/cm^2^. At 24, 72, and 144 h of semen storage, sperm kinematics were recorded by computer-assisted semen analysis (CASA), and sperm membrane integrity, mitochondrial activity, and DNA integrity were evaluated by flow cytometry (Section 2.6).

#### 2.2.4. Experiment 4: Effect of Ambient Light on Sperm Quality After PDI_blue_ Compared to PDI_white_

The aim was to verify the hypothesis that semen samples with a lower TMPyP concentration (used with PDI_blue_) are less susceptible to sperm damage compared to higher TMPyP concentration (used with PDI_white_) when exposed to ambient room light after illumination. Semen from 10 boars, extended in Androstar Premium, was subjected to PDI_blue_ with 0.5 µM TMPyP and 2.5 mW/cm^2^, and to PDI_white_ with 2 µM TMPyP and 4.5 mW/cm^2^. Thereafter, samples were exposed to laboratory room light for 3 h. The room light sources (Fa. Philips, Hamburg Germany, CoreLine Panel RC133V G5 36S/840 PSU W62L62 NOC) were mounted on the ceiling of the laboratory and each lamp had a dimension of 61 mm height, 620 mm width, and 620 mm depth. The lamps had a luminous flux value of 3600 lumens and a radiation angle of 120°. The light had a neutral white light color. The samples were placed on a table at a distance of approximately 1.65 m from the light source. The light intensity reaching the samples was 487.1 ± 16.8 lux, which was measured by a chromameter (8040-218, Fa. Minolta, Camera CO., LTD., Osaka 541, Japan). At 2, 72, and 144 h, sperm kinematics and membrane integrity were assessed (Section 2.6.1 and Section 2.6.2).

### 2.3. Chemicals and Media

All chemicals used in the experiments were of analytical grade. The chemicals were purchased from Merck KGaA (Darmstadt, Germany), Sigma-Aldrich Productions GmbH (Steinheim, Germany), Carl Roth GmbH &Co. KG (Karlsruhe, Germany), Minitüb GmbH (Tiefenbach, Germany), and Polysciences (Europe GmbH, Hirschberg an der Bergstraße, Germany). The TMPyP was purchased from TCI Chemicals (Tokyo, Japan). The antibiotic-free Androstar Premium (APrem) extender was obtained from Minitüb GmbH (Tiefenbach, Germany). It contains a cell shield-protecting component and an organic bactericidal supplement, designed for long-term semen storage (Minitüb GmbH; Ref. 13533/7001; (Minitüb, 2024)). Semen extenders used for microbiology experiments were free of conventional antibiotics and were sterile-filtered before use.

### 2.4. Semen Collection, Sample Preparation, and Storage

Semen was collected from 10 different boars, aged 1 to 6 years, belonging to the breeds Piétrain, Large White, and Landrace. The boars were housed on straw in individual pens at the Unit for Reproductive Medicine, University of Veterinary Medicine Hannover, and were treated in accordance with the European Commission Directive for Pig Welfare following the ARRIVE guidelines. Entire ejaculates without the bulbourethral gland secretion were routinely collected once a week by trained personnel using the gloved hand method. All procedures involving animals were approved by the Animal Welfare Committee of the University of Veterinary Medicine Hannover. Normospermic ejaculates with at least 70% sperm motility and less than 25% morphological abnormalities were diluted in one step with a prewarmed (35 °C) extender medium to 20 × 10^6^ sperm/mL. After storage for 90 min at room temperature, subsamples with volumes of 8 mL were filled in transparent semen bags described in Section 2.1. The PS TMPyP was added, and the bags were sealed. For each experimental day, a separate sample bag was prepared. Samples were subjected to PDI, and control samples without TMPyP were kept in the dark. Thereafter, samples were stored up to 144 h in the dark at 17 °C in a temperature-controlled climate cabinet. Any handling and examination of the semen samples containing TMPyP was performed in a darkened room at room temperature.

### 2.5. Microbiology

The bacterial counts were assessed from a 10-fold serial dilution with sterile PBS, ranging from 10^−1^ to 10^−10^ depending on the expected bacterial load. Volumes of 100 µL of the diluted samples were plated on Columbia agar plates with sheep blood. The agar plates were incubated at 37 °C (Model 100-800, Fa. Memmert GmbH + Co. KG, Schwabach, Germany) for 24 h to 48 h under aerobic conditions. Thereafter, the bacterial colonies were counted, and the colony-forming units per milliliter (CFU/mL) were calculated. Agar plates were evaluated if 10–300 colonies had grown on the plate. Bacterial species in raw semen were identified by MALDI-TOF MS (microFlex LT, Bruker Daltonics GmbH & Co. KG, Bremen, Germany) using the software Biotyper (Bruker Daltonics GmbH & Co. KG, Server Version 4.1.100). The bacterial isolates were stored at −80 °C and then cultured on sheep blood agar for 24 h at 35 °C under aerobic conditions. For Experiment 2, semen was spiked with *E. coli* isolated from boar semen.

### 2.6. Spermatology

#### 2.6.1. Sperm Kinematics

Computer-assisted semen analysis (CASA) using the system AndroVision^®^ (Version 1.2, Minitüb GmbH, Tiefenbach, Germany) equipped with a TV adapter (60-C 1″ 1.0×, Carl Zeiss Microscopy GmbH, Jena, Germany), a digital camera (acA2440–75uc, Basler AG, Ahrensburg, Germany), and a heated automatic scan stage was employed to assess sperm kinematics [20]. Briefly, 2 mL semen samples were prewarmed in a water bath at 38 °C for 30 min under air. Subsamples were then filled into 20 µL chambers (Leja Products, B.V., Nieuw Vennep, The Netherlands), and at least 500 spermatozoa in 5 successive fields were analyzed at a rate of 75 pictures/s. Spermatozoa were classified as “motile” when the curved-line velocity (VCL) exceeded 24 μm/s and the amplitude of lateral head displacement was greater than 1 μm. Progressively motile spermatozoa were identified by a VCL exceeding 41 μm/s and a velocity straight line (VSL) greater than 15 μm/s. The kinematic parameters, including total motility (%), progressive motility (%), VCL (μm/s), amplitude of lateral head displacement (ALH; μm), and beat cross frequency (BCF; Hz), were determined [21].

#### 2.6.2. Sperm Membrane Integrity and Mitochondrial Activity

A flow cytometer (Cyto Flex, Beckman Coulter GmbH, Krefeld, Germany) equipped with three lasers (488 nm, 50 mW, 638 nm, 50 mW, 405 nm, 80 mW) was used to assess the integrity of sperm membranes and mitochondrial activity, as described previously [3]. Briefly, for the assessment of acrosome and plasma membrane integrity, samples containing TMPyP were stained with final concentrations of 1.3 μM Hoechst (H) 33342, and 2 μM fluorescein-conjugated peanut agglutinin (FITC-PNA). Control samples were stained with final concentrations of 1.3 μM H 33342, 1.5 μM propidium iodide (PI), and 2 μM FITC-PNA. The TMPyP and PI are membrane-impermeable stains with similar emission spectra and were used as a viability stain. Hoechst 33342 was detected on fluorescence detector PB-450 (450/45 nm BP), FITC-PNA on the FITC channel (525/40 nm BP), PI on the PC5.5 channel (690/50 nm BP), and TMPyP on the BV650 channel (660 nm/20 BP). Non-DNA-containing particles were identified by negative H 33342 staining and were excluded from the analysis. Spermatozoa with intact membranes were negative for the stains of PI/TMPyP and FITC-PNA.

Mitochondrial activity was assessed after a 20-min incubation at 38 °C in HBS containing final concentrations of 2.7 μM H 33342, 0.003 μM rhodamine (Rh) 123, and 0.19 mmol/L PI. Rhodamine was detected on the FITC channel (525/40 nm BP). Viable sperm with mitochondrial activity was determined when negative for the PI stain and positive for Rh123.

#### 2.6.3. DNA Fragmentation

The DNA fragmentation index (DFI) was evaluated with the Sperm Chromatin Structure Assay (SCSA; [22]). Two hours post-illumination, the extended semen samples were washed in sterile phosphate-buffered-saline (PBS) and centrifuged at 70 g for 10 min to remove TMPyP. This procedure was repeated twice. Thereafter, the extended semen samples were snap-frozen. Post-thawing, the samples were treated as previously described [23] with few modifications. All media used in the SCSA were kept light-protected and chilled on ice throughout the experiment. Aliquots (20 µL) of the thawed semen were diluted with 180 µL TNE buffer (consisting of 0.15 M NaCl, 0.01 M TRIS-HCL, 1 mM Na_2_-EDTA; pH 7.4). To this, was added 400 µL of an acid detergent solution (consisting of 0.15 M NaCl, 0.1% Triton-X100, 0.08 M HCl; pH 1.2). The solution was mixed and incubated on ice for 30 s. Thereafter, a quantity of 1.2 mL of the Acridine Orange staining solution was added (consisting of 6 μg/mL Acridine Orange, 0.0037 M citric acid, 0.126 M Na_2_HPO_4_, 0.1 mmol/L Na_2_ EDTA, 0.15 M NaCl; pH 6.0). Following an incubation time of 2.5 min, the sample was mixed once more and measured with the flow cytometer. A Gallios flow cytometer (Fa. Beckman Coulter) equipped with a 488 nm laser (22 mW) for excitation and filters for the detection of green fluorescence (525/40 nm) and red fluorescence (620/30 nm) was used. The software Gallios Cytometry List Mode Data Acquisition & Analysis Software (Version 1.2) was used for implementing and gating the collected data. The results were then analyzed with the software FCSExpress (Version 3). Each sample underwent analysis of 10,000 events. For every 10 samples measured, a reference sample with a known DFI was also evaluated to serve as a control. Every semen sample was measured twice; the DFI was determined and the mean value of both measurements for each sample was calculated. When AO enters the cell and encounters normal double-stranded DNA (stable chromatin), green fluorescence is detected. If the DNA is compromised and thus single-stranded (denatured chromatin), the fluorescence is red [22]. The DFI indicates the ratio of red fluorescence to the total fluorescence (red and green) [24].

### 2.7. Statistical Analysis

Data analysis was conducted with IBM SPSS Professional software (Version 29.0.0.0 (241); SPSS Inc., IBM, Armonk, NY, USA). Initially, the data were assessed for normal distribution both graphically, using a histogram with a normal distribution curve, and analytically, using the Shapiro–Wilk Test. Normally distributed data related to sperm motility, membrane integrity, mitochondrial activity, and DNA fragmentation were analyzed using a two-way ANOVA. If the samples were assessed on different examination days, the two-way ANOVA with repeated measurements was employed. When the spermatological data were not normally distributed, the Friedman Test was implemented. Results were corrected by Bonferroni.

Bacterial count data were analyzed using the Friedman Test. Differences among samples were assessed within storage time points and were considered significant if the *p*-value was below the threshold level of 0.05. Spermatological data are presented as mean ± standard deviation (SD), whereas microbiology data are displayed as mean ± standard error of the mean (SEM).

## 3. Results

### 3.1. Experiment 1: Effect of Blue LED Light on Sperm Kinematics

**Figure 3:**
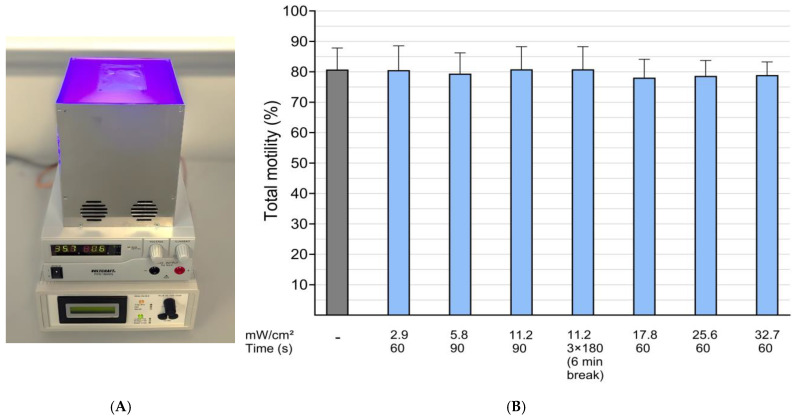
Illumination of semen samples with blue LED light. Experimental setup (**A**): from top to bottom: lamp with sample bag (5.2 × 7 cm), power supply, and programmable current controller. Effect of blue LED light on sperm motility in stored boar semen samples (**B**): dose-response of illumination in nine extended semen samples from three different boars. After illumination, samples were stored at 17 °C in the dark. Data were combined from analysis at 24, 72, and 144 h of storage. Data are shown as mean ± SD. Values did not differ (*p* > 0.05); Experiment 1.

This dose-response study using illumination intensities up to 32.7 mW/cm^2^ for 60 s demonstrated no effects on sperm motility in comparison to dark controls (*p* > 0.05). All the illuminated samples had a total motility exceeding 75% (Figure 3B). There was no effect on sperm kinematic parameters, including progressive motility, VCL, BCF, and ALH (Table 1).

### 3.2. Experiment 2: Antimicrobial Efficiency of PDI_blue_ Compared to PDI_white_

**Figure 4:**
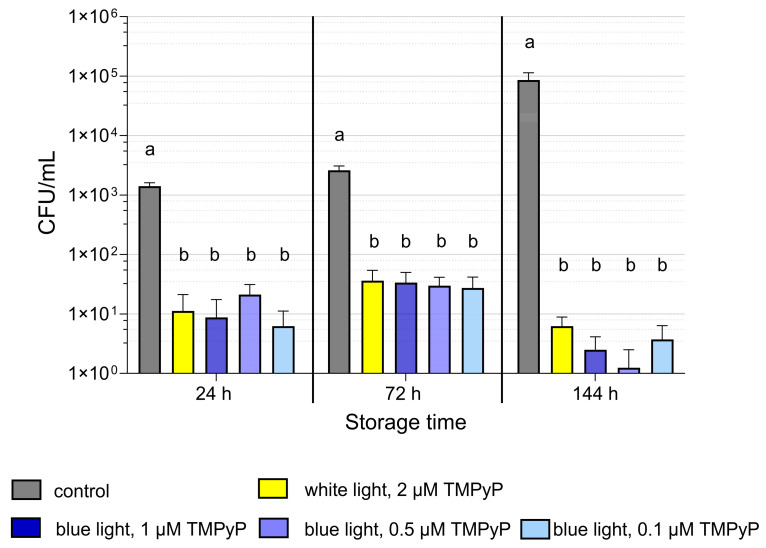
Bacterial counts (CFU/mL) after photodynamic inactivation (PDI) in extended boar semen using white (PDI_white_) or blue (PDI_blue_) LED light with different concentrations of the photosensitizer TMPyP. Semen samples (n = 8) were spiked with *E. coli* to reach an initial bacterial count of 1.6 × 10³ CFU/mL. The PDI samples were illuminated for 90 s. In PDI_white_, samples were illuminated with an intensity of 4.5 mW/cm². In PDI_blue_, different intensities were used, depending on the TMPyP concentration: 1 µM TMPyP and 1.2 mW/cm², 0.5 µM TMPyP and 2.5 mW/cm², 0.1 µM TMPyP and 10.6 mW/cm². Thereafter, all samples were stored up to 144 h at 17 °C in the dark. Bacterial counts were determined at three time points during semen storage. Data are shown as mean ± SEM. a,b: different letters indicate differences among values within storage time (*p* < 0.05); Experiment 2.

The naturally occurring seminal bacteria flora cultured in blood agar comprised seven gram-negative and five gram-positive bacteria (Table 2). Bacterial counts in the raw semen ranged between 1.1 × 10^3^ and 4.6 × 10^4^ CFU/mL, with a mean value of 1.7 × 10^3^ CFU/mL. Bacterial counts after PDI_blue_ in extended semen containing three different TMPyP concentrations (up to 1.0 µM) showed no significant difference to each other and the samples containing 2 µM TMPyP with PDI_white_ (*p* > 0.05; Figure 4). All the illuminated samples exhibited a further decline of bacterial loads, reaching counts below the detection limit (<10^1^ CFU/mL) after 144 h of storage. The reduction in CFU was 4 log levels compared to controls at the end of semen storage.

PDI_blue_ with 0.5 µM TMPyP and an illumination intensity of 2.5 mW/cm² recorded the lowest values for bacterial counts after 144 h of semen storage. Consequently, this method was chosen for subsequent experiments.

### 3.3. Experiment 3: Sperm Quality After PDI_blue_ Compared to PDI_white_

**Figure 5:**
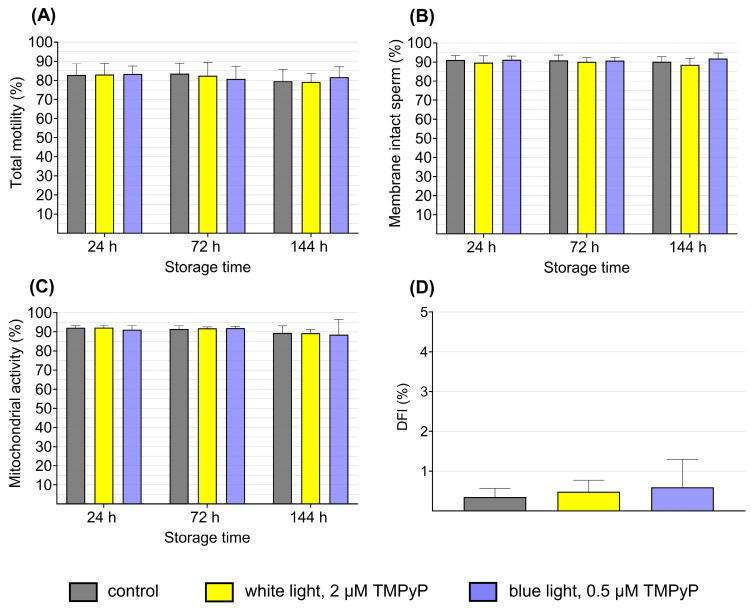
Sperm quality after photodynamic treatment with low TMPyP concentration (PDI_blue_) compared to higher TMPyP concentration (PDI_white_). The extended semen samples (n = 8 boars) were illuminated for 90 s with 2.5 mW/cm² in PDI_blue_ and 4.5 mW/cm^2^ in PDI_white_. After illumination, the semen was stored up to 144 h at 17 °C in the dark. Motility (**A**), Membrane integrity (**B**), Mitochondrial activity in viable sperm (**C**), and the DNA fragmentation index (DFI) at 2 h (**D**) are shown. Values within storage times did not differ (*p* > 0.05). Data are shown as means ± SD; Experiment 3.

The effect of PDI_blue_ compared to PDI_white_ in long-term stored semen is depicted in Figure 5. Throughout the duration of storage, no difference in the sperm quality characteristics was observed, neither within PDI–treated samples nor in comparison to the dark controls (*p* > 0.05). Motility (total motile sperm; Figure 5A) remained at a consistently high level (around 80%) throughout the storage period. Progressive motility and other sperm kinematic traits, presented in Table 3, also remained high without notable differences among the sample groups.

The membrane integrity, analyzed as the percentage of sperm with intact plasma membranes (“viable sperm”) and intact acrosome, remained at a consistently high level, ranging between 88.4 and 91.8% throughout semen storage. No significant differences were found among the two PDI groups and the controls (Figure 5B).

The percentage of viable spermatozoa with active mitochondria remained high during semen storage at levels exceeding 84.5%. No discernible differences were observed among the three sample groups (*p* > 0.05; Figure 5C).

The results of the SCSA indicated no significant influence of the semen treatment on the integrity of the sperm DNA. The DFI values were all below 1% across all groups and demonstrated no differences among each other (Figure 5D).

### 3.4. Experiment 4: Effect of Ambient Light on Sperm Quality After PDI_blue_ Compared to PDI_white_

**Figure 6:**
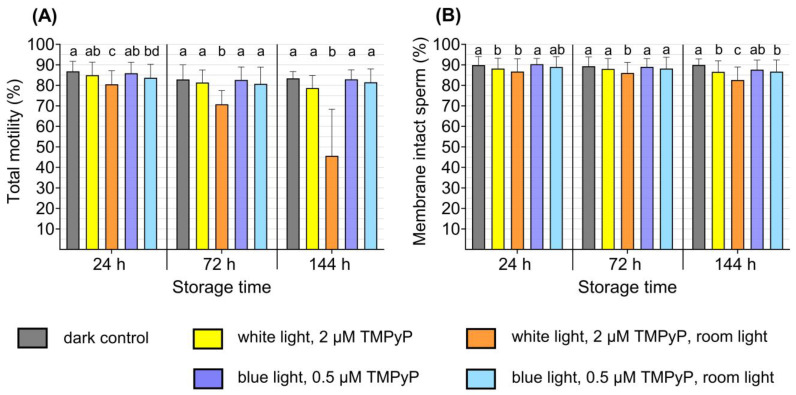
Effect of 3 h room light exposure after the photodynamic treatment of boar semen samples with blue LED light and low TMPyP concentration (PDI_blue_) compared to white LED light with higher TMPyP concentration (PDI_white_). Motility (**A**), and membrane integrity (**B**) are shown. The extended semen samples (n = 10 boars) were illuminated for 90 s using an intensity of 2.5 mW/cm^2^ in PDI_blue_ and 4.5 mW/cm^2^ in PDI_white_. After PDI, or after PDI and exposure to room light, semen was stored in the dark at 17 °C for up to 144 h. Data are shown as mean ± SD at three time points of semen storage. a–c: different letters indicate differences among values within storage time (*p* < 0.05); Experiment 4.

Exposure to room light following PDI_blue_ did not affect sperm motility, when compared to dark controls with and without TMPyP, at any storage time points. The PDI_blue_ samples maintained high motility values (>80%) until the end of semen storage, regardless of whether the semen tubes were subjected to room light. In contrast, PDI_white_ samples exposed to room light exhibited a significant reduction in motility compared to the dark controls as early as 24 h of storage (Figure 6A). Over the course of 144 h of storage, motility in the PDI_white_ samples exposed to room light dropped further to 45.6% ± 21.6%, with an average 33.1% decrease in comparison to the dark controls. The sperm kinematic parameters progressive motility, velocity and BCF were lower in PDI_white_ samples following room light exposure as compared to the dark samples, particularly after 144 h of storage. Room light exposure had no impact on sperm kinematics in PDI_blue_ samples (Table 4).

In samples exposed to room light after PDI_blue_, a high value of 87.6% ± 4.4% membrane intact spermatozoa was detected after long-term storage (144 h), which did not differ from PDI_blue_ samples kept in the dark (86.7% ± 5.3%). Conversely, PDI_white_ samples, when exposed to room light, showed lower percentages of membrane-intact spermatozoa at 72 and 144 h when compared to their dark controls (Figure 6B).

## 4. Discussion

This study demonstrates that the modified PDI technology utilizing a low PS concentration and blue LED light can be applied to extended boar semen without causing undesired post-illuminary photoreactions in ambient laboratory light. This methodology is a crucial progression towards practical application in artificial insemination laboratories, where alternatives to antibiotics in semen extenders are urgently needed [1,2]. Common contemporary laboratory room light sources are white LED lamps, similar to the light source previously established for PDI in boar semen [3] and used for PDI_white_ in this study. The similarity of light sources poses a risk for additional photoreactions during sample handling in room light. In contrast, using an excitation source that overlaps the Soret band of TMPyP, compared to normal laboratory light, which nearly does not, opens the option to reduce the sensitivity to unwanted light exposure by reducing the TMPyP concentration. Uncontrolled photostimulation during sample handling in room light may affect semen quality, especially when the spermatozoa are additionally stressed by long-term storage. Even though the absorption of photons by the porphyrin TMPyP during exposure to ambient light is much less efficient compared to the absorption during illumination, it is still sufficient to initiate photoeffects due to a certain overlap with the spectrum of TMPyP [25]. Handling semen samples containing TMPyP in ambient light consequently induces the production of cytotoxic ROS, especially singlet oxygen. These ROS target not only bacteria, but also off-target biomolecules [26], including polyunsaturated fatty acids, plasmalogens, and sphingomyelins present in membranes of mammalian spermatozoa [27]. Excessive oxidative stress may thus affect not only sperm membranes [28], but can also induce lesions in ATP utilization or the contractile apparatus of the flagellum, leading to a reduction in motility [29]. In the present study, the risk for unwanted light effects was diminished by a 75% reduction in the TMPyP concentration compared to the original PDI_white_ protocol [3]. This was achieved by replacing the white light excitation source with blue LED light, which allows for more selective illumination in the Soret band range of TMPyP.

Any change of the illumination source in the PDI protocol must consider that light, even in the absence of exogenous PS, may induce photophysical and photochemical reactions through the activation of intracellular PS in the spermatozoa. Blue light in the range of 400–500 nm can either enhance or decrease sperm motility [16,17]. Negative effects on ram sperm motility were observed with blue light at 360 nm, 1.5 mW/cm^2^, and 5 min illumination [30], whereas sperm exposure to 410 nm laser light, 0.67 mW/cm^2^ for 60 s improved sperm motility [31]. In contrast, blue LED light illumination with 470 nm, 5 mW/cm^2^ for 3 min has been considered as motility-stimulating low-level light therapy (LLLT) through the acceleration of mitochondrial respiration and ATP synthesis in cases of asthenozoospermia in men [32]. Our study using blue LED light with 415 nm up to 32 mW/cm^2^ for 60 s did not influence sperm motility in the stored semen. It is worth noting that the first examination was at 2 h after illumination, whereas in the cited studies in rams and humans, sperm motility was already assessed within 30 min after illumination. Thus, short-term effects of blue LED light on boar sperm kinematics, similar to those reported for red light [33], cannot be excluded in our study, but probably would not be relevant for fertility, where the semen is used for insemination at least several hours after processing.

The light effect on spermatozoa as well as on bacteria is mediated by endogenous PS, among which porphyrins are also present [34]. This explains that blue light itself has antimicrobial activity [35]. However, the low amount and different types of intracellular PS in bacteria require high light doses to reach antimicrobial efficiency [35], which precludes the application in semen. Photo-mediated effects against bacteria are more efficient in the presence of exogenous PS. PDI using the PS curcumin (up to 20 µM) and blue light is applied in food preservation [36,37], and PDI with 10 µM TMPyP for 120 s with 20 mW/cm^2^ showed antimicrobial efficacy in a model for human root canal tooth [38]. In the liquid suspension of extended semen examined in the present study, low TMPyP concentrations (<1 µM) and low light intensity (<10.6 mW/cm^2^) were antimicrobially effective. A high broad-spectrum antibacterial effect was obtained by reducing the growth of different species of gram-positive (n = 5) and gram-negative bacteria (n = 6, predominantly *E. coli*) to a similarly low level in all tested PDI regimes. This result was expected because the different PDI regimes generated an equal amount of highly reactive singlet oxygen, which is the key molecule initiating the PDI effect [39]. Obviously, the different sources of light (blue vs. white) and TMPyP concentrations (0.5 vs. 2 µM) did not influence the antibacterial efficiency. All tested PDI patterns decreased the bacterial load during long-term storage by 5 log_10_ levels to values below 10 CFU/mL. This is a value far below the reported threshold levels between 10^3^ and 10^7^ CFU/mL for the occurrence of detrimental effects on semen quality [40,41,42,43,44,45].

The main challenge in using PDI for the decontamination of semen is the maintenance of the sperm’s morphological and functional integrity. This was successful with the original PDI_white_ protocol [3], and likewise with the modified PDI_blue_ established in this study. As outlined above, this is not surprising due to the formation of equal quantities of singlet oxygen. Outer sperm membranes, such as bacterial membranes, are negatively charged and may also be targeted by the cationic TMPyP as a result. The mechanism underlying the selective effectiveness of PDI towards bacteria remains unknown but may reside in the dissimilarity of the outer bacterial cell wall compared to that of spermatozoa. Differences in membrane composition, architecture, and organization between the two cell types could favor the electrostatic interaction of TMPyP with bacteria. Alternatively, spermatozoa, being approximately 100-fold larger than bacteria, might be more resistant to PDI effects due to the relatively lower exposure of their cell surface to the singlet oxygen molecules in the semen suspension. Nonetheless, exposing sperm to light always raises concerns about DNA damage. Due to its shorter wavelength, blue light carries higher energy compared to white light. To date, no information on the effect of blue light (with or without PS) on sperm DNA integrity has been available [17]. Our study shows that PDI with blue LED light, like PDI with white light, in an antimicrobially effective dosage does not increase DNA fragmentation in boar spermatozoa. In contrast to classical radiation therapy or exposure to ionizing radiation, the risk for gene mutations caused by PDI appears to be low. Moderate photodynamic treatment as used here may induce cell damage resulting from oxidative injury and does not directly induce genetic mutations. Noteworthy, in a proof of principle in vivo trial conducted in 2023, healthy offspring were born using PDI-semen [3], and no clinical symptoms for mutagenesis were reported during their lifetime. Nonetheless, when further trying the new technology in insemination trials, the health of the offspring should be monitored.

The sperm characteristics examined here are limited to in vitro data. Based on the encouraging results from the investigation of sensitive sperm structures and function, such as kinematics and membrane integrity (also termed “viability”), as well as mitochondrial activity, maintenance of fertilization ability after PDI_blue_ seems likely. Before confirmation with insemination trials, further adaptation of the PDI technology to practical use is required. An important step towards this goal was to enhance the light tolerance of PDI-semen by the reduction in the TMPyP concentration in the sample. Finally, the initial hypothesis underlying this study was confirmed in our last experiment, where the beneficial effects of PDI_blue_ compared to PDI_white_ on the functional integrity of the boar spermatozoa under typical laboratory light conditions were shown.

## 5. Conclusions

A PDI illumination pattern with blue LED was established, which proved to maintain high semen quality at concomitant broad-spectrum antibacterial efficacy. The illumination with blue LED light is clearly advantageous over PDI with white LED light, as it allows the use of significantly lower photosensitizer concentrations. This increases the resistance to ambient light and thus protects spermatozoa from additional, uncontrolled photostimulation. The main advantages of the PDI approach for replacing antibiotics are that it is eco-friendly and does not cause resistance. Further adaption of the innovative technology before use in larger insemination trials is necessary. This comprises, in particular, the effective illumination of larger sample sizes corresponding to boar semen portions used for insemination. Another direction for future research is to explore the full potential of this technology for inactivating different kinds of potential pathogens in boar semen. From a technical and economic perspective, the application of PDI in boar semen processing in AI centers is a realistic option because the illumination in an LED tunnel would take only seconds, and the TMPyP is of low cost. Overall, the present study provides an important step towards the adaptation of PDI technology for practical use in semen preservation, aiming to enhance the sustainability of pig reproduction.

## Figures and Tables

**Table 1:** Effect of blue LED light on sperm kinematics in stored boar semen samples. Dose-response of illumination in nine extended semen samples from three different boars. After illumination, samples were stored at 17 °C in the dark. Data were combined from analysis at 24, 72, and 144 h of storage. There were no significant differences among the samples (*p* > 0.05); data are shown as mean ± SD; Experiment 1.

Illumination Regime	Parameter			
	Progressive Motility (%)	Velocity Curvilinear Line VCL (µm/s)	Beat Cross Frequency BCF (Hz)	Amplitude of Lateral Head Displacement ALH (µm)
Dark control	77.2 ± 7.8	159 ± 20	28.7 ± 2.3	1.3 ± 0.1
2.9 mW/cm^2^, 60 s	77.0 ± 7.9	156 ± 17	29.7 ± 1.2	1.3 ± 0.1
5.8 mW/cm^2^, 90 s	75.3 ± 7.3	154 ± 16	29.9 ± 1.5	1.2 ± 0.1
11.2 mW/cm^2^, 90 s	77.6 ± 7.9	158 ± 17	29.4 ± 1.7	1.3 ± 0.1
11.2 mW/cm^2^, 3 × 180 s (6 min break)	77.2 ± 8.3	155 ± 18	28.8 ± 2.2	1.2 ± 0.1
17.8 mW/cm^2^, 60 s	73.9 ± 6.6	154 ± 17	29.0 ± 1.5	1.2 ± 0.1
25.6 mW/cm^2^, 60 s	74.8 ± 5.2	157 ± 16	29.4 ± 1.8	1.2 ± 0.1
32.7 mW/cm^2^, 60 s	74.8 ± 5.2	155 ± 17	29.6 ± 2.2	1.2 ± 0.1

**Table 2:** Bacterial counts (CFU/mL) and bacterial species in the raw semen after culture in sheep blood agar. Dilution of the raw semen reduced the bacterial counts by approximately one log level. N = 9 boars; Experiment 2.

	Mean	SD	Min	Max
**Bacterial Count** (CFU/mL)	1.53 × 10^4^	1.34 × 10^3^	1.1 × 10^3^	4.6 × 10^4^
	**Bacterial species**			
Gram-negative	*Pseudomonas aeruginosa*; *Providencia stuartii*; anhemolytic *Escherichia coli*; *Klebsiella pneuomoniae*; *Citrobacter koseri*; *Pantoea agglomerans*; non-fermenter			
Gram-positive	*Staphylococcus* (coagulase-negative); *Coryneform bacteria*; *Enterococcus faecalis*; *Streptococcus* (ß-hemolytic); *Bacillus* species			

**Table 3:** Sperm kinematics after photodynamic treatment of boar semen with blue light LED and low TMPyP concentration (PDI_blue_) compared to white LED light with higher TMPyP concentration (PDI_white_). The extended semen samples (n = 8 boars) were illuminated for 90 s using an intensity of 4.5 mW/cm^2^ in PDI_white_ and 2.5 mW/cm^2^ in PDI_blue_. The semen was stored after PDI up to 144 h at 17 °C in the dark. Data show means and SDs at three time points of storage. Values within a storage time did not differ (*p* > 0.05); Experiment 3.

Parameter	Treatment	24 h	72 h	144 h
Progressive motility (%)	dark control	76.1 ± 6.8	74.7 ± 9.2	73.6 ± 6.7
	white LED, 2 µM TMPyP	79.4 ± 7.1	77.8 ± 9.0	72.9 ± 5.9
	blue LED, 0.5 µM TMPyP	79.7 ± 5.6	76.4 ± 8.2	75.7 ± 7.0
Velocity curvilinear line VCL (µm/s)	dark control	120 ± 18	132 ± 18	174 ± 19
	white LED, 2 µM TMPyP	151 ± 22	155 ± 18	173 ± 20
	blue LED, 0.5 µM TMPyP	154 ± 23	155 ± 25	172 ± 21
Beat cross frequency BCF (Hz)	dark control	32.6 ± 2.0	30.7 ± 1.6	26.6 ± 2.0
	white LED, 2 µM TMPyP	30.6 ± 2.6	30.2 ± 1.7	27.0 ± 1.9
	blue LED, 0.5 µM TMPyP	30.8 ± 2.5	30.3 ± 2.7	28.5 ± 2.2
Amplitude of lateral head displacement ALH (µm)	dark control	1.0 ± 0.1	1.1 ± 0.1	1.4 ± 0.2
	white LED, 2 µM TMPyP	1.2 ± 0.2	1.3 ± 0.1	1.4 ± 0.2
	blue LED, 0.5 µM TMPyP	1.2 ± 0.2	1.2 ± 0.2	1.4 ± 0.2

**Table 4:** Effect of 3 h room light exposure after the photodynamic treatment of boar semen samples with blue light LED and low TMPyP concentration (PDI_blue_) compared to white LED light with higher TMPyP concentration (PDI_white_). The extended semen samples (n = 10 boars) were illuminated for 90 s using an intensity of 2.5 mW/cm² in PDI_blue_ and in 4.5 mW/cm² in PDI_white_. After PDI or after PDI and exposure to room light, semen was stored in the dark at 17 °C for up to 144 h. Data show means and SD at three time points of semen storage. a,b: Different letters indicate differences between values within storage time (*p* < 0.05); Experiment 4.

Parameter	Treatment	24 h	72 h	144 h
Progressive motility (%)	dark control	78.3 ± 9.9 ^a^	74.4 ± 11.8 ^a^	74.3 ± 8.8 ^a^
	white LED, 2 µM TMPyP	77.5 ± 9.3 ^a^	72.6 ± 9.4 ^a^	65.7 ± 9.0 ^b^
	white LED, 2 µM TMPyP, room light	72.5 ± 9.5 ^b^	58.0 ± 10.5 ^b^	30.5 ± 20.0 ^c^
	blue LED, 0.5 µM TMPyP	78.3 ± 9.0 ^a^	73.4 ± 8.6 ^a^	72.3 ± 9.6 ^ad^
	blue LED, 0.5 µM TMPyP, room light	76.3 ± 9.6 ^a^	71.2 ± 10.2 ^a^	69.7 ± 10.2 ^bd^
Velocity curvilinear line VCL (µm/s)	dark control	172 ± 25 ^a^	155 ± 20 ^a^	165 ± 24 ^ab^
	white LED, 2 µM TMPyP,	151 ± 19 ^a^	151 ± 22 ^a^	162 ± 27 ^ab^
	white LED, 2 µM TMPyP, room light	130 ± 19 ^b^	147 ± 25 ^a^	132 ± 45 ^a^
	blue LED, 0.5 µM TMPyP	157 ± 28 ^a^	155 ± 17 ^a^	165 ± 24 ^ab^
	blue LED, 0.5 µM TMPyP, room light	148 ± 19 ^ab^	150 ± 24 ^a^	168 ± 28 ^b^
Beat cross frequency BCF (Hz)	dark control	29.8 ± 2.4 ^a^	28.1 ± 2.4 ^a^	25.5 ± 2.4 ^a^
	white LED, 2 µM TMPyP	29.9 ± 1.7 ^a^	27.3 ± 2.6 ^a^	24.7 ± 3.1 ^a^
	white LED, 2 µM TMPyP, room light	28.4 ± 3.2 ^a^	24.2 ± 3.9 ^b^	20.1 ± 2.5 ^b^
	blue LED, 0.5 µM TMPyP	29.1 ± 2.2 ^a^	27.3 ± 2.7 ^a^	25.1 ± 2.6 ^a^
	blue LED, 0.5 µM TMPyP, room light	29.5 ± 2.2 ^a^	27.7 ± 3.0 ^a^	24.6 ± 2.5 ^a^
Amplitude of lateral head displacement ALH (µm)	dark control	1.3 ± 0.2 ^a^	1.2 ± 0.1 ^a^	1.3 ± 0.2 ^a^
	white LED, 2 µM TMPyP,	1.2 ± 0.1 ^a^	1.2 ± 0.2 ^a^	1.3 ± 0.2 ^a^
	white LED, 2 µM TMPyP, room light	1.0 ± 0.1 ^b^	1.2 ± 0.2 ^a^	1.2 ± 0.3 ^a^
	blue LED, 0.5 µM TMPyP	1.2 ± 0.2 ^a^	1.2 ± 0.1 ^a^	1.3 ± 0.2 ^a^
	blue LED, 0.5 µM TMPyP, room light	1.2 ± 0.1 ^ab^	1.2 ± 0.2 ^a^	1.3 ± 0.2 ^a^

## Data Availability

The study data are present in the main text. For further inquiries, please contact the corresponding author.

## Abbreviations

The following abbreviations are used in this manuscript:

AI	Artificial insemination
LED	Light-emitting diode
PDI	Photodynamic inactivation
